# HIF-1α: a bridge connecting sepsis and acute respiratory distress syndrome

**DOI:** 10.1186/s40001-025-03107-z

**Published:** 2025-08-31

**Authors:** Shi-Yan Liu, Hang Ruan, Shu-sheng Li

**Affiliations:** 1 Department of Critical-Care Medicine, Tongji Hospital, Tongji Medical College, Huazhong University of Science and Technology, 1095# Jiefang Ave, Wuhan, 430030 China; 2 Department of Emergency Medicine, Tongji Hospital, Tongji Medical College, Huazhong University of Science and Technology, Wuhan, China

**Keywords:** Sepsis, Acute respiratory distress syndrome, Critical care, HIF-1α, Hypoxia, Molecular medicine

## Abstract

Sepsis is a life-threatening condition marked by an abnormal host response to infection that can result in organ dysfunction, making it recognized as one of the primary causes of acute respiratory distress syndrome (ARDS). The pathophysiology of sepsis involves a cascade of events, including heightened pulmonary capillary permeability, dysfunction of alveolar epithelial cells, and the infiltration of inflammatory cells, such as neutrophils, macrophages, monocytes, and lymphocytes. The presence of these inflammatory cells triggers capillary leakages, alveolar epithelial damage, and the accumulation of fluid within the alveolar spaces, leading to compromised gas exchange, acute respiratory failure, and the progression to ARDS. In this complex scenario, Hypoxia-Inducible Factor-1α (HIF-1α) emerges as a pivotal player in maintaining cellular oxygen homeostasis and responding to hypoxia and inflammatory stimuli. This narrative review delves into the intricate molecular and biological characteristics of HIF-1α, elucidating its regulatory role within the context of sepsis and ARDS. By exploring the therapeutic potential of targeting HIF-1α, this review seeks to offer valuable insights into the underlying mechanisms linking sepsis to ARDS. Ultimately, this exploration of HIF-1α seeks to enhance our comprehension of sepsis pathogenesis, identify novel therapeutic avenues, and lay a strong theoretical groundwork for future clinical interventions.

## Background

Sepsis is a systemic inflammatory disorder triggered by a severe infection, capable of impacting multiple organs across the body [1]. Among the organs vulnerable to its effects, the lungs are particularly at risk, as individuals often develop acute lung injury/acute respiratory distress syndrome (ALI/ARDS) in this condition [2]. ALI/ARDS precipitated by sepsis represents a profound complication of sepsis, prominently marked by intractable hypoxemia, respiratory compromise, and non-cardiac pulmonary edema. Compared to non-sepsis-associated ARDS, sepsis-associated ARDS is characterized by greater disease severity and higher mortality [3]. Consequently, identifying the key factors and elucidating the molecular mechanisms underlying ARDS development in sepsis patients has been a long-standing research hotspot in critical care medicine.

Hypoxia-inducible factor-1α (HIF-1α) is a crucial transcription factor with a wide array of target genes, including nearly 100 genes related to hypoxia adaptation, inflammation development, and tumor growth [4]. The pathological changes in sepsis, characterized by dysregulation of proinflammatory and anti-inflammatory responses, dysfunction in macrocirculation and microcirculation, endothelial damage, inflammatory exudation, and tissue/cellular hypoxia, are fundamentally linked to alterations in HIF-1α levels in septic patients [5]. Clinical studies confirm a significant elevation in HIF-1α expression and plasma levels in septic patients compared to non-septic individuals [6]. Similarly, numerous studies have identified HIF-1α as a key factor in the development of ALI and ARDS. These findings suggest that HIF-1α may be a critical molecular link between sepsis and ARDS [7, 8]. However, there is currently a lack of comprehensive studies delineating the role of HIF-1α in sepsis and ARDS.

In previous investigations, our research has encompassed a range of studies examining the involvement and functionality of HIF-1α in sepsis[6, 9, 10]. Drawing upon this prior research, the current study delves into the pivotal role of HIF-1α as a key connector between sepsis and ARDS. Moreover, the study consolidates insights on potential pharmaceutical interventions targeting HIF-1α.

## Molecular biology characterization and function of HIF-1α

HIF-1 is comprised of two subunits: the constitutively expressed β-subunit (ARNT) and the oxygen-sensitive α-subunit, of which there are three isoforms—HIF-1α, HIF-2α, and HIF-3α[11–13]. Belonging to the basic helix–loop–helix-per-ARNT-Sim (bHLH–PAS) protein family, HIF-1α features a bHLH domain and two PAS domains (PAS-A and PAS-B) crucial for α and β subunit heterodimerization and DNA binding[14]. Alongside the bHLH and PAS domains, HIF-1α possesses two transactivation domains: the N-terminal transactivation domain (N-TAD) and the C-terminal transactivation domain (C-TAD), along with an oxygen-dependent degradation domain (ODDD), with a notable overlap between the N-TAD and ODDD [9]. The N-TAD functions significantly in HIF-1α stabilization and degradation prevention [15], while the C-TAD primarily enables coactivator interactions like CBP/p300 to modulate the transcriptional function of HIF-1α [16, 17]. HIF-1α is involved in regulating pathways related to metabolic adaptation, erythropoiesis, angiogenesis, vascular tone, cell growth, differentiation, cell survival, and apoptosis.

**Fig. 1:**
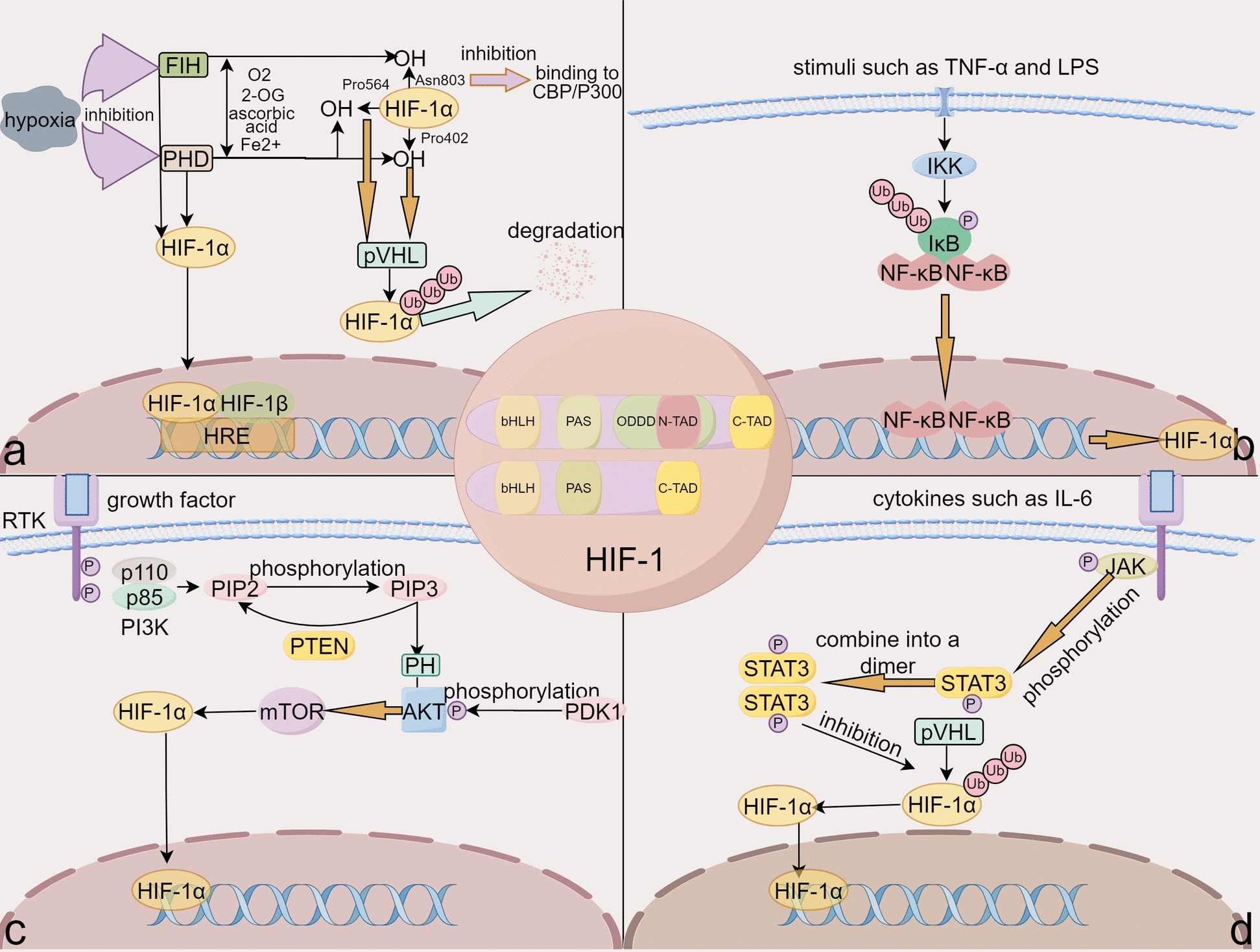
Regulation of HIF-1α: oxygen-dependent and inflammation-related pathways. **a** Oxygen-dependent pathway regulates HIF-1α levels. Under normoxic conditions, prolyl hydroxylases (PHDs) and factor inhibiting HIF (FIH) hydroxylate specific proline and asparagine residues of HIF-1α. This modification marks HIF-1α for degradation via a chain of biological processes. However, under hypoxic conditions, it inhibits the activity of PHDs and FIH, stabilizing HIF-1α. This stabilized form translocates to the nucleus, where it dimerizes with HIF-1β to form the HIF complex, which then binds to HREs in the regulatory regions of target genes. **b** Nuclear factor-κB (NF-κB) pathway regulates HIF-1α levels. Activation of inhibitor of kappa B kinase (IKK) leads to phosphorylation and subsequent ubiquitination (Ub) of IκB (Inhibitor of NF-κB) protein, releasing the NF-κB dimer. The free NF-κB translocates to the nucleus, binds DNA, and promotes the transcription of genes that regulate HIF-1α. **c** Phosphatidylinositol 3-kinase/protein kinase B (PI3K/Akt) pathway regulates HIF-1α levels. Factor stimulation activates PI3K, generating inositol triphosphate (PIP3), a second messenger that activates Akt. Akt subsequently activates the mammalian Target of Rapamycin (mTOR), which contributes to HIF-1α regulation. **d** Signal transducer and activator of transcription 3 (STAT3) pathway regulates HIF-1α levels. Cytokine-mediated activation of JAK phosphorylates STAT3. The phosphorylated STAT3 forms dimers that compete with the von Hippel–Lindau (pVHL) for binding to HIF-1α, thereby influencing its levels. List of abbreviations: *HIF-1α:* Hypoxia Inducible Factor-1, *PHDs:* Prolyl hydroxylases, *FIH:* Factor inhibiting *HIF*, *HREs:* Hypoxia response elements, *NF-κB:* Nuclear Factor-κB, *IKK:* Inhibitor of kappa B kinase, *Ub:* Ubiquitination, *IκB:* Inhibitor of NF-κB, *DNA:* Deoxyribonucleic acid, *PI3K/Akt:* Phosphatidylinositol 3-kinase/protein kinase B, *PIP3:* Inositol triphosphate, *mTOR:* Mammalian target of rapamycin, *STAT3:* Signal transducer and activator of transcription 3, *JAK:* Janus kinase, *pVHL:* von Hippel–Lindau.

The level of HIF-1α is regulated by both oxygen-dependent and non-oxygen-dependent mechanisms. Under normoxic conditions, proline and asparagine residues in HIF-1α are hydroxylated, leading to its degradation through a series of biological processes [18, 19]. Under non-oxygen-dependent conditions, inflammatory factors can regulate HIF-1α levels [20]. Figure 1 shows the regulation of HIF-1α levels by oxygen-dependent pathways and inflammation-related pathways.

Under normoxic conditions, HIF-1α is rapidly degraded within 5 min [21]. The degradation occurs mainly through two pathways. The primary pathway involves the hydroxylation of two proline residues (Pro402 and Pro564) within the ODDD of HIF-1α by prolyl hydroxylases (PHDs), which marks HIF-1α for recognition by the VHL protein to facilitate its binding to the E3 ubiquitin ligase complex. The complex then polyubiquitinates HIF-1α to degrade it by the 26S proteasome [22, 23]. In addition, a secondary degradation pathway involves the hydroxylation of an asparagine residue (Asn803) in HIF-1α by factor inhibiting HIF (FIH). This modification prevents the C-TAD of HIF-1α from binding to coactivators, thereby promoting HIF-1α degradation [18, 24].

In addition, HIF-1α is regulated by multiple non-oxygen-dependent pathways, including the NF-κB, STAT3, and PI3K/Akt signaling pathway. NF-κB serves a key role in regulating immune and inflammatory reactions by binding to pro-inflammatory cytokines, chemokines, enzymes generating inflammatory mediators, and adhesion molecules [25]. The study suggests that the upregulation of HIF-1α during acute inflammation is interconnected with NF-κB activity [26]. In the context of sepsis, inflammatory factors such as tumor necrosis factor-α (TNF-α) and interleukin-1β (IL-1β) modulate HIF-1α expression by activating the NF-κB pathway [20, 27]. Acting as a key driver, NF-κB, a central modulator of HIF-1α accumulation [28], directly binds to the HIF-1α promoter to enhance its transcription and expression [29]. This interaction enhances HIF-1α-mediated processes, such as glycolytic metabolism and angiogenic factor production [30]. Furthermore, STAT3 contributes to the stabilization of HIF-1α protein levels by impeding the interaction between pVHL and HIF-1α, thereby reducing HIF-1α degradation [31]. Inflammatory factors, such as IL-6 and IL-15, can impact HIF-1α expression by activating the STAT3 pathway [32, 33]. The T-cell receptor (TCR) modulates HIF-1α through STAT3 signaling, leading to substantial accumulation of both HIF-1α mRNA and protein [34]. The PI3K/Akt pathway plays a crucial role in boosting HIF-1α protein levels by enhancing translation, independent of its effect on HIF-1α mRNA levels [35]. Furthermore, the MAPK pathway, and the Mdm2 pathway also hold significance in the regulation of HIF-1α.

## Dual role of HIF-1α in cellular perspective

The etiology of ARDS is typically classified into primary direct lung injury and secondary indirect lung injury. Indirect lung injury, which includes causes, such as sepsis, pancreatitis, burns, and medications, is a common contributor to ARDS. Sepsis is recognized as a major cause of ARDS, accounting for 32% of ARDS etiology [36]. In a statistical analysis of 3,022 patients with acute respiratory distress syndrome (ARDS) in intensive care units across 50 countries, 484 cases of extrapulmonary sepsis were identified, accounting for approximately 16%. However, sepsis also involves pulmonary factors, so the proportion of ARDS cases caused by sepsis should be greater than 16% [37]. In addition to the hypoxia directly induced by ARDS, sepsis can also trigger tissue hypoxia and ischemia due to endothelial damage, resulting in relative hypovolemia, decreased vascular tone, and microcirculatory dysfunction [38]. The excessive release of inflammatory mediators during sepsis can upregulate the expression levels of HIF-1α [39–41]. Thus, HIF-1α plays a crucial role in coordinating physiological responses to hypoxic and inflammatory stimuli. The primary mechanisms by which sepsis-associated ARDS occurs include an inflammatory response and cytokine storm, excessive immune cell infiltration and activation, oxidative stress, increased vascular permeability, and dysfunction of the vascular endothelium [5].

### HIF-1α contributes to sepsis-associated ARDS by modulating endothelial function

The endothelium, comprising endothelial cells, is recognized as the largest functional tissue in the human body, exerting various critical functions such as serving as a crucial physical barrier separating blood and tissues, regulating blood flow dynamics, synthesizing and releasing vasoactive substances, and being receptive to a myriad of bioactive molecules [42]. According to the classification based on endothelial function, endothelial dysfunction can be categorized into barrier dysfunction, and endothelial permeability. Sepsis-induced endothelial damage can result in relative hypovolemia, decreased vascular tone, microcirculatory dysfunction, and subsequent tissue ischemia and hypoxia. In addition, this endothelial injury can trigger the activation of the coagulation system, leading to microthrombosis, pulmonary edema, and pulmonary hemorrhage, exacerbating systemic hypoxia [2].

Protracted and severe endothelial barrier dysfunction can result in compromised microcirculatory blood flow, inadequate tissue perfusion, and potentially life-threatening organ failure [6]. Consequently, endothelial barrier dysfunction is a significant contributor to organ dysfunction in sepsis. HIF-1α modulates vascular endothelial cell barrier function through diverse mechanisms. Previous research has delved into the impacts of HIF-1α on endothelial function. Studies have demonstrated that HIF-1α levels rise in endothelial cells under hypoxic and inflammatory conditions, influencing the expression of downstream target genes [43]. In animal models, HIF-1α has been shown to elevate endothelin-1 secretion and disrupt endothelial cell tight junctions in a burn rat model, resulting in increased endothelial cell permeability in burn rats [44]. Mice lacking HIF-1α display impaired vascular repair, persistent inflammatory responses, and elevated mortality [45]. In cellular studies, HIF-1α is known to modulate endothelial cell function and immune responses through the forkhead box M1 (FoxM1) signaling pathway [46]. Consequently, HIF-1α plays a crucial role in the impairment of endothelial barrier function.

The endothelial system also plays a crucial role in controlling vascular permeability, regulating the extravasation of solutes, fluids, large molecules, hormones, and blood cells. Endothelial permeability, a selective mechanism that facilitates exchange between blood vessels, tissues, and organs [47], is closely associated with ARDS, which is characterized by increased pulmonary vascular permeability [48]. HIF-1⍺ mediates the transcription of various angiogenic factors, such as vascular endothelial growth factor (VEGF) and angiopoietin (Ang), and promotes their transcription [49], which in turn leads to vascular endothelial cell expansion to increase vascular permeability and fibrinogen extravasation [50]. VEGF, a key mediator in airway inflammatory diseases, is strongly correlated with airway inflammation. In addition, it can exacerbate vascular leakage and amplify inflammatory responses in septic patients. Overexpression of VEGF has also been linked to impaired lung development and structural damage in adult lungs, increasing lung injury, and further contributing to ARDS progression [51].

### HIF-1α involvement in sepsis-induced ARDS by safeguarding lung epithelial cells

**Fig. 2:**
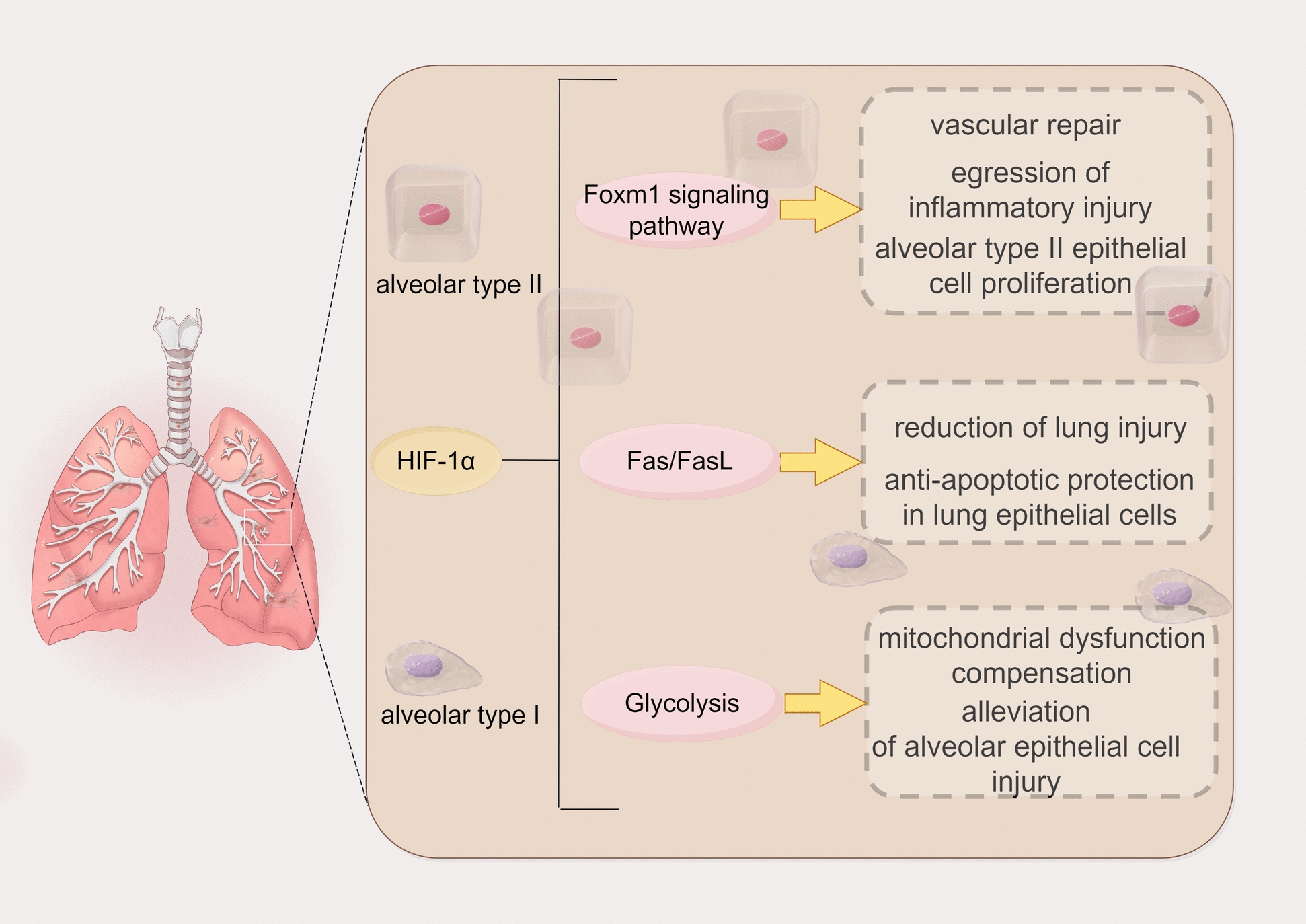
Multifaceted protective mechanisms of HIF-1α in pulmonary epithelial cells. HIF-1α promotes vascular repair, egression of inflammatory injury, and alveolar type II epithelial cell proliferation by regulating the Foxm1 signaling pathway. HIF-1α promotes reduction of lung injury and anti-apoptotic protection in lung epithelial cells by regulating the Fas/FasL. HIF-1α alleviates alveolar epithelial cell injury by enhancing glycolytic activity and compensating for mitochondrial dysfunction. List of abbreviations: *HIF-1α:* Hypoxia Inducible Factor-1

In the context of sepsis-associated ARDS, lung epithelial cells play a fundamental role. Injury to these cells disrupts the alveolar capillary barrier, leading to diffuse pulmonary edema and worsening ARDS. Furthermore, the infiltration of immune cells in the lungs triggers inflammatory responses by releasing proteases and cytokines, aggravating damage to pulmonary vascular endothelial cells and alveolar epithelial cells [52, 53]. This process increases capillary permeability, disrupts the alveolar capillary barrier, and results in fluid leakage into the alveolar spaces, ultimately precipitating diffuse pulmonary edema. Moreover, elevated levels of reactive oxygen species (ROS) directly impair pulmonary vascular endothelial cells and alveolar epithelial cells due to oxidative stress, impairing gas exchange and contributing to ARDS progression [54]. In essence, these multifaceted pathways culminate in lung injury induced by sepsis, characterized by alveolar epithelial cell damage and subsequent tissue and cellular hypoxia. Hypoxia inhibits the degradation of HIF-1⍺, causing its accumulation and subsequent translocation to the nucleus, where it forms a dimer with HIF-1β to activate the transcription of downstream target genes [55]. In sepsis, pathogen activation of the immune system leads to a massive release of inflammatory mediators such as cytokines (TNF-α, IL-1β, and IL-6) from immune cells [52], which initiate an autocrine/paracrine cycle leading to excessive cytokine release, which further leads to a cytokine storm production, resulting in a further increase in the inflammatory response and exacerbation of inflammation and injury in the lungs [56]. Studies have shown that HIF-1α can promote vascular repair, regression of inflammatory injury, and stimulation of alveolar type II epithelial cell proliferation by modulating the HIF-1α/Foxm1 signaling pathway [45, 57]. Furthermore, it safeguards lung epithelial cells against Fas/FasL-mediated apoptosis, reducing lung injury in mice [58]. Notably, by enhancing glycolytic activity and compensating for mitochondrial dysfunction, HIF-1α mitigates alveolar epithelial cell injury and improves arterial oxygenation [59]. Figure 2 shows the multifaceted protective mechanisms of HIF-1α in pulmonary epithelial cells.

### HIF-1α contributes to sepsis-associated ARDS by modulating immune cell function

HIF-1α is closely associated with immune cells and plays a vital role in their response to pathological stress and the adaptive regulation of environmental changes. HIF-1⍺ plays a crucial role in regulating immune cell function by modulating apoptosis, survival, differentiation, and immune responses, thereby influencing sepsis-associated ARDS. In monocytes, macrophages, and neutrophils, HIF-1⍺ is essential for maintaining cellular energy homeostasis and is associated with processes, such as cell aggregation, invasion, and motility [60]. In addition, HIF-1⍺ is pivotal in adaptive immunity, affecting T cell proliferation, differentiation, metabolism, and Th17/Treg balance. It also contributes to B cell development and autoimmunity. In neutrophils, HIF-1⍺ significantly affects function by reducing apoptosis, increasing degranulation, promoting ROS production, and producing oxidizing enzymes that kill organisms such as *Staphylococcus aureus* [61]. HIF-1⍺ regulates survival in hypoxic environments by inhibiting apoptosis, leading to the accumulation of activated neutrophils [62]. This accumulation exacerbates lung endothelial and epithelial damage [63]. Therefore, elevated HIF-1⍺ leads to decreased neutrophil apoptosis, which in turn affects neutrophil function and increases the number of neutrophils, further promoting lung endothelial and epithelial injury and ARDS development. M1 macrophages, which are key in the first line of defense against bacterial infections, are mainly found in hypoxic environments and need to rely on glycolysis produced by HIF-1⍺ to produce adenosine triphosphate (ATP). Moreover, the activation of M1 macrophages can also be rapidly induced when glycolysis produced by HIF-1⍺ is present. In M1 macrophages, HIF-1⍺ can lead to sustained production of the inflammatory factor IL-1β, resulting in further exacerbation of the inflammatory response [64], worsening sepsis-associated acute lung injury (SALI), and contributing to ARDS progression[53]. Table 1 shows the regulatory effects of HIF-1α levels on different immune cells.

**Table 1:** Regulatory impact of HIF-1α levels on diverse immunocyte subtypes

Cell types	Role of HIF-1⍺	Biological function	Main mechanisms	Ref
Monocyte	Reprogrammed to an immunosuppressive phenotype	Metabolic reprogramming	Upregulates IRAKM	[65, 66]
Neutrophil	Regulation of neutrophil survival	Immune cell function	Through induction of NF-κB, it leads to PHD3 expression	[62]
Macrophage	Affecting ATP production and macrophage function	Metabolic reprogramming	Through glycolysis	[67, 68]
Mast cells	Promoting inflammatory factor production, angiogenesis, and increasing MCETs	Angiogenesis	Not given	[69, 70]
DC	Affecting migration, interferon production, and T cell activation	Metabolic reprogramming and immune cell function	Leads to metabolic reprogramming to glycolysis for DC migration	[71, 72]
T cell	Inhibiting T cell proliferation under ET	Immune cell function	Upregulates PD-L1 expression	[73, 74]
T cell	Influencing on pro-inflammatory cytokines and antimicrobial function	Immune cell function	Inhibits NF-κB activation by blocking p50 transcription	[75]
T cell	Affecting T cell differentiation and metabolism	Immune cell function	Not given	[34, 76]
T cell	Regulation of Th17/Treg balance	Immune cell function	HIF-1α activates RORγt or interacts with RORγt through p300 recruitment and histone acetylation	[77]
B cell	Affecting differentiation, development, survival, and mediated autoimmunity and transformation	Immune cell function	In BM, HIF-1α changes dynamically during B-cell development	[78]
B cell	Regulation of CD11b in B cells	Immune cell function	Affects CD11b binding to the HRE region of the Itgam promoter	[79]

*List of abbreviations: Ref*.: Reference, *IRAKM:* Interleukin-1 receptor-associated kinase M, *PHD3:* Prolyl hydroxylase3, *MCETs:* Mast cell extracellular trap, *DC:* Dendritic cell, *ET:* Endotoxin tolerance, *PD-L1:* Programmed death-ligand 1, *BM:* Bone marrow, *HRE:* Hypoxia response element

### HIF-1α involvement in sepsis-associated ARDS through inhibiting mitochondrial function

Mitochondrial dysfunction is a key pathophysiological mechanism underlying sepsis-associated ARDS [80]. Mitochondria play a pivotal role in cellular synthesis and metabolic processes, acting as the central hub for material metabolism and energy conversion in the human body [81]. Impairment of mitochondrial function disrupts normal cellular activity, resulting in tissue and organ damage. Mitochondrial dysfunction is a critical driver of sepsis progression, caused by factors, such as the generation of reactive nitrogen and oxygen species, suppression of mitochondrial autophagy, changes in mitochondrial dynamics, and alterations in mitochondrial membrane permeability [82]. It was shown that under hypoxic conditions HIF-1α can protect cells from mitochondrial dysfunction by affecting mitochondrial function through a dual mechanism: on one hand, it reduces ROS production under hypoxic conditions by increasing mitochondrial autophagy and regulating the expression of cytochrome oxidase subunits, which in turn reduces oxidative stress-induced cell death [83]; On the other hand, HIF-1α can undergo mitochondrial translocation and prevent oxidative stress-induced apoptosis [84]. At the level of molecular regulation, HIF-1α has been recognized as a key regulator of HO-1 [85]. Heme oxygenase-1 (HO-1), an enzyme known for its anti-inflammatory, antioxidant, and cytoprotective effects, exerts these benefits by breaking down heme into carbon monoxide (CO), iron, and biliverdin, which is then converted into bilirubin [86]. Experimental evidence suggests that elevated HO-1 provides a strong cytoprotective effect in the lungs by degrading heme, and also attenuates sepsis-induced ALI by reducing Golgi and endoplasmic reticulum stress [87]. Studies have demonstrated that endotoxin-induced ALI can be ameliorated by modulating the HIF-1α/HO-1 signaling pathway, which also helps maintain the dynamic balance of mitochondrial fusion and fission, as observed in treatments using dexmedetomidine (DEX) [88].

## Dual role of HIF-1α in molecular perspective

### HIF-1α contributes to sepsis-associated ARDS by modulating inflammatory factors

**Fig. 3:**
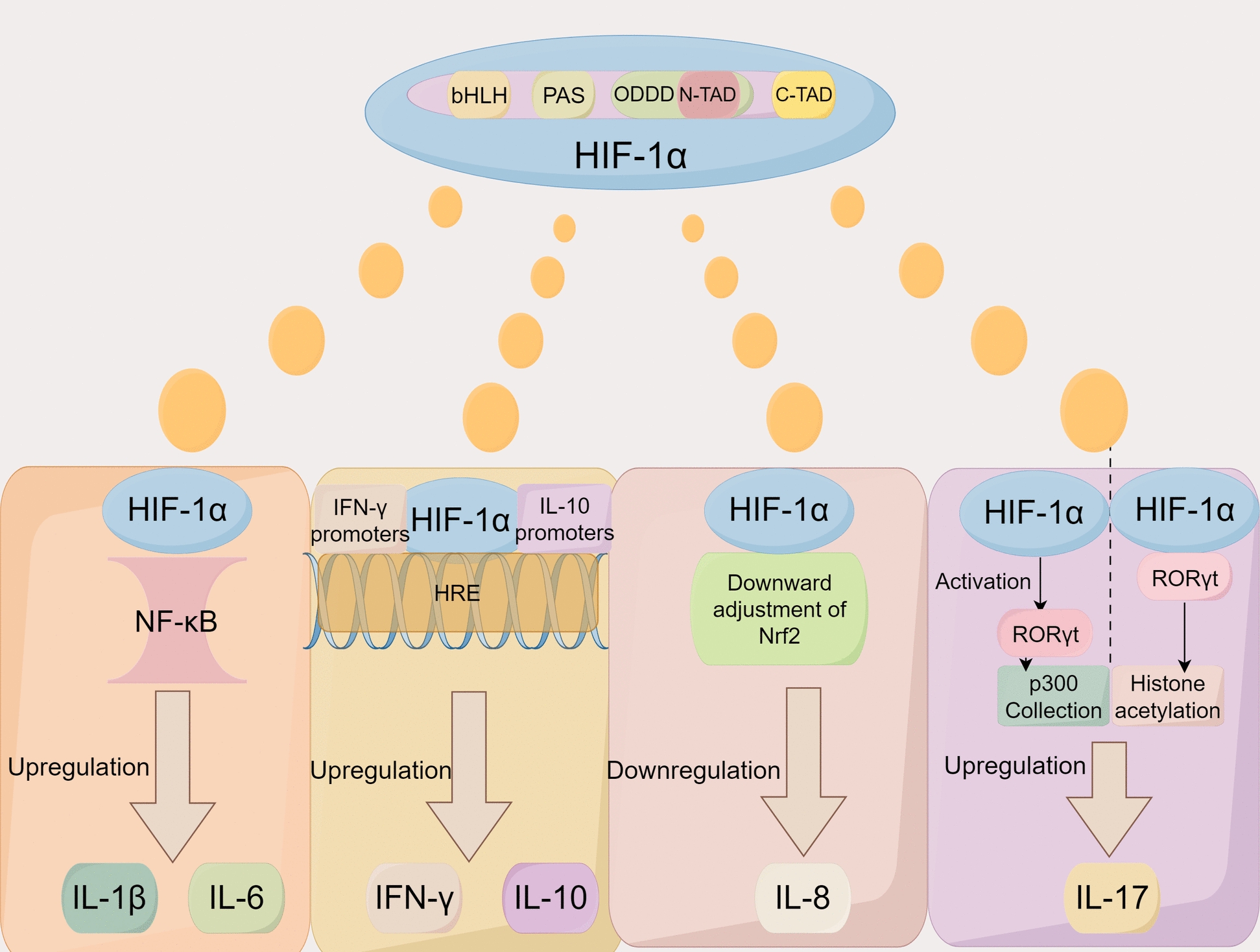
Regulatory Role of HIF-1α on Key Inflammatory Factors. HIF-1α upregulates IL-1β and IL-6 through the NF-κB signaling pathway; HIF-1α binds to the IL-10 and IFN-γ promoters at the HRE to upregulate IL-10 and IFN-γ; Inhibition of IL-8 expression by HIF-1α is associated with downregulation of nuclear factor erythroid 2-related factor 2 (Nrf2) By activation of retinoid-related orphan nuclear receptor γt (RORγt), or in conjunction with RORγt, HIF-1α upregulates IL-17 through p300 recruitment and histone acetylation. List of abbreviations: *HIF-1α:* Hypoxia Inducible Factor-1, *IL-1β:* Interleukin-1β, *IL-6:* Interleukin-6, *NF-κB:* Nuclear Factor-κB, *IL-10:* Interleukin-10, *IFN-γ:* Interferon-γ, *HRE:* Hypoxia response elements, *IL-8:* Interleukin-8, *Nrf2:* Nuclear factor erythroid 2-related factor 2, *RORγt:* Retinoid-related orphan nuclear receptor γt, *IL-17:* Interleukin-17.

Inflammation plays an important role in sepsis-associated ARDS. Sepsis is initiated by the complement system and specific cell surface receptors detecting various infection-derived microbial products and endogenous danger signals concurrently, leading to an inflammatory imbalance. Pathogenic micro-organisms infiltrate the system, releasing pathogen-associated molecular patterns (PAMPs) that invoke immune reactions, prompting immune cells to discharge an array of inflammatory mediators, initiating a cytokine storm, and orchestrating immune system activation [9]. Moreover, injuries such as sepsis, trauma, and burns can prompt the release of endogenous pattern recognition receptor agonists known as damage-associated molecular patterns (DAMPs), subsequently triggering inflammatory responses. The excessive inflammatory response is related to the release of inflammatory cytokines, including TNF-α, IL-1β, IL-12, and IL-18 [89], which play pivotal roles in the development of the cytokine storm. These cytokines activate NF-κB expression, initiating an autocrine/paracrine cycle that leads to excessive release of inflammatory factors, thereby producing cytokine storms. This process intensifies the inflammatory response, contributes to lung inflammation and injury [56], and ultimately leads to ARDS. HIF-1α was found to influence prognosis by regulating inflammatory factors and thus prognosis. In the inflammatory response, HIF-1α can modulate it by modulating a variety of inflammatory factors involved in the development of sepsis [90]. HIF-1α has been demonstrated to enhance the expression of TNF-α. In myeloid cells, HIF-1α regulates TNF-α production through NO dependent mechanisms [91]. Furthermore, IL-1β is directly regulated by HIF-1α, and stabilization of HIF-1α promotes IL-1β production. As observed in LPS-induced succinate accumulation, it enhances IL-1β protein expression [92]. In hypoxic conditions, MG-132 inhibits the degradation of HIF-1α, leading to increased IL-1β release in astrocytes [93]. Experiments involving the intraperitoneal injection of HIF-1α activators and inhibitors in mice revealed that a reduction in HIF-1α levels activates TLR4 expression [94]. Figure 3 summarizes the regulation of HIF-1α for major inflammatory factors.

### HIF-1α involvement in sepsis-associated ARDS through metabolic reprogramming

Metabolic reprogramming is the cellular response to environmental stimuli, including cell activation, proliferation, and differentiation, which entails alterations in metabolism-related enzymes, metabolites, and metabolic pathways. In sepsis, a wide range of cell types experience significant alterations in metabolic patterns, transitioning from oxidative phosphorylation (OXPHOS) to glycolysis. The occurrence of mitochondrial dysfunction during sepsis results in bioenergetic collapse, a critical aspect contributing to the initiation of multi-organ failure [95]. However, upregulation of HIF-1α promotes glycolysis, compensates for energy deficits caused by mitochondrial dysfunction, prevents ATP depletion, and protects the pulmonary epithelium from energy failure, thereby mitigating the risk of sepsis-associated ARDS [59]. Furthermore, when elevated HIF-1α induces a metabolic shift towards glycolysis, it results in increased lactate production [96]. Lactate, produced by alveolar type II (ATII) cells, has been shown to enhance the anti-inflammatory activity of alveolar macrophages (AM) and suppress excessive inflammation in ALI. A study using a mouse model of ALI demonstrated that administering lactic acid to the local airways reduced pro-inflammatory cytokines, such as IL-1 and IL-6, in AM while simultaneously increasing anti-inflammatory factors such as IL-10. In addition, lactic acid attenuated inflammatory responses triggered by LPS stimulation [97]. Thus, elevated HIF-1α not only mitigates energy failure but also suppresses inflammation, reducing the incidence of sepsis-associated ARDS. Tissue-resident alveolar macrophages (TR-AM), located in the alveolar lumen, mainly maintain lung homeostasis under normal conditions [98]. HIF-1α promotes hypoxia adaptation and enhances glycolysis in TR-AM, enabling their metabolic adaptation to the hypoxic microenvironment. This adaptation prevents TR-AM death, enhances their survival, and sustains their function despite mitochondrial inhibition. Consequently, the stabilization of TR-AM reduces pro-inflammatory cytokine production in the lungs.

## Potential drugs targeting HIF-1α

HIF-1α has become an important biomarker for the diagnosis, prognosis, and treatment of various diseases due to its regulatory role in hypoxia, metabolism, and other processes. In the field of oncology, numerous clinical studies have demonstrated that the overexpression of HIF-1α in tumors is significantly associated with tumor size, metastasis, and poor prognosis [99, 100]. For example, high levels of HIF-1α expression are associated with poorer survival outcomes [101]; HIF-1α overexpression is also associated with tumor size, tumor stage, lymph node metastasis, and overall survival [102]. A study of five patients treated with Belzutifan, a new HIF-α inhibitor, found that this new HIF-α inhibitor is an effective and well-tolerated treatment option for patients with von Hippel–Lindau syndrome-associated renal cell carcinoma, hemangioblastoma, and sporadic advanced pretreated renal cell carcinoma [103]. Furthermore, HIF-1α, a key regulator in sepsis and ARDS, has been recognized as a potential target for developing therapies to treat these conditions. Its pharmacological roles encompass the regulation of inflammatory mediators, immune cell function, angiogenesis, vascular permeability, metabolic reprogramming, and cell death. However, it has been found that in sepsis-associated ARDS, upregulation or down-regulation of HIF-1α leads to the same endpoints seemingly contradictory, such as reducing oxidative stress, but the mechanism behind it involves a complex mechanism of action. Upregulation of HIF-1α induces an increase in the expression of glycolytic enzymes, which promotes cellular shifts towards glycolytic metabolism and maintenance of bioenergetic homeostasis [83], and thus may reduce the level of oxidative stress. Meanwhile, HIF-1α elevation under hypoxic conditions increases mitochondrial autophagy and regulates the expression of cytochrome oxidase subunits to reduce ROS production under hypoxic conditions, which in turn reduces oxidative stress-induced cell death to further protect hypoxic cells [83]. In contrast, HIF-1α down-regulation may reduce the over-activation of M1 macrophages and neutrophils by inhibiting the activation of pathways such as NF-κB [62, 64], which reduces the inflammatory response and may affect the level of oxidative stress. Bidirectional regulation of HIF-1α in sepsis-associated ARDS reflects the body's dynamic adaptation to hypoxia and inflammation. Table 2 compiles drugs that have been identified to both downregulate and upregulate HIF-1α in the management of sepsis or ARDS.

**Table 2:** Potential drug for regulating HIF-1α levels in the management of sepsis or ARDS

Drug	Regulation	Biological function	Main mechanisms	Ref
Lidocaine	Down	Inhibiting glycolysis and reducing inflammation	Inhibits of NF-κB signaling	[104]
Resveratrol	Down	Promoting vasodilatory function	Inhibits of Rac-1 and HIF-1α and upregulation of eNOS expression	[105]
mSPAM nanoassembly	Down	Anti-inflammatory	Suppresses HIF-1α expression in LPS-stimulated macrophages	[106]
2-methoxyestradiol	Down	Reducing organ damage	Reduces HIF-1α	[107]
Xanthoxylin	Down	Anti-inflammatory	Downregulates the Akt/HIF-1α/NF-κB pathway	[108]
MEL	Down	Reducing inflammation and improving lung damage	Downregulates the HIF-1α/GLUT1/NLRP3 pathway	[109]
Cynaroside	Down	Preventing macrophage polarization to a pro-inflammatory phenotype	Targeting the PKM2/HIF-1α axis inhibits glycolysis-related proteins including HIF-1α	[110]
TIIA	Down	Reducing inflammation	Inhibits HIF-1α protein translation and promotes HIF-1α protein degradation	[111]
Norisoboldine	Down	Inhibiting inflammatory response and promoting macrophage M2 polarization	Activates PKM2 and attenuates HIF-1α expression	[112]
PFCs	Down	Reducing inflammation	Reduces hypoxemia, which attenuates HIF-1α	[56]
Propofol	Down	Inhibition of lung epithelial cell injury	Inhibits HIF-1α expression	[113]
Erlotinib	Down	Inhibition of M1 polarization, promotion of M2 polarization, and attenuation of sepsis-induced ALI	Inhibits the PKM2/HIF-1α pathway	[114]
Emodin	Down	Reducing ALI	Inhibits mTOR/HIF-1α/VEGF pathway	[115]
FG-4592	Up	Reducing ALI	Inhibition of PHD stabilizes HIF-1α, which in turn induces TR-AM metabolic reprogramming and reduces susceptibility to mitochondrial function inhibition	[98]
DMOG	Up	Reducing inflammation and protecting intestinal barrier function	Inhibition of PHD upregulates HIF-1α to reduce inflammatory factors, oxidative stress intestinal permeability, and pathological damage	[116]
DEX	Up	Treatment of LPS-induced ALI	Up-regulation of HIF-1α/HO-1 maintains a dynamic balance of mitochondrial fusion/fission	[88]
Shenfu Injection	Up	Reducing sepsis-induced lung injury	Upregulates HIF-1α to mediate mitochondrial autophagy and improve mitochondrial function	[117]
Rabeprazole	Up	Reducing sepsis-induced lung injury	Promotes vascular repair through endothelial HIF-1α/FoxM1 signaling	[46]

*List of abbreviations: Ref*.: Reference, *Rac-1:* Ras-related C3 botulinum toxin substrate 1, *eNOS:* endothelial nitric oxide synthase, *GLUT1:* glucose transporter type 1, *NLRP3:* NOD-like receptor protein 3, *FoxM1:* Forkhead box protein M 1

The down-regulation of HIF-1α can reduce various organ dysfunctions caused by sepsis, as well as treat or alleviate sepsis through anti-inflammation, inhibition of glycolysis, and promotion of vasodilatation, and improve ARDS by reducing apoptosis and oxidative stress, decreasing inflammatory cytokines, and promoting epithelial cell as well as vascular repair. Drugs designed to down-regulate HIF-1α can be classified according to their specific mechanisms of action [9], including (1) promoting HIF-1α degradation, (2) inhibiting its DNA transcription and expression, (3) blocking mRNA translation, (4) preventing HIF-1α binding to HREs, (5) interfering with HIF-1α transcriptional complex formation. For instance, tanshinone IIA (TIIA) reduces inflammation and LPS-induced lung injury by inhibiting HIF-1α protein translation and promoting its degradation.

The upregulation of HIF-1α can be demonstrated efficacy in ameliorating symptoms of ARDS and sepsis through mechanisms, such as attenuating inflammatory factor expression, reducing oxidative stress, facilitating metabolic reprogramming, and modulating mitochondrial biogenesis. PHD inhibitors, a type of drug that boosts HIF-1α levels by inhibiting PHD activity, fall within the category of medications that enhance HIF-1α expression. Furthermore, a separate class of drugs enhances therapeutic outcomes in ARDS and sepsis by stabilizing HIF-1α, thereby augmenting its protective functions.

## Conclusion

In this study, HIF-1α was identified as a key factor influencing the development and prognosis of sepsis-associated ARDS, demonstrating dual roles in both promoting and mitigating disease progression. Future research should focus on elucidating the specific molecular mechanisms and pathways through which HIF-1α contributes to the pathogenesis of sepsis-associated ARDS.

## Data Availability

No datasets were generated or analysed during the current study.

## Abbreviations

2-OG: 2-Oxoglutarate
A2BR: A2B receptor
ADP: Adenosine diphosphate
AK: Adenosine kinase
ALI: Acute lung injury
AM: Alveolar macrophages
Ang: Angiopoietin
ARDS: Acute respiratory distress syndrome
ATF4: Activating transcription factor 4
ATII: Alveolar type II
ATP: Adenosine triphosphate
BALF: Bronchoalveolar lavage fluid
bHLH-PAS: Basic-Helix-Loop-Helix-per-ARNT-Sim
CARS: Compensatory anti-inflammatory response syndrome
CO: Carbon monoxide
C-TAD: C-transactivation domain
DEX: Dexmedetomidine
DNA: Deoxyribonucleic acid
ET: Endotoxin tolerance
FIH: Factor inhibiting HIF
HIF: Hypoxia Inducible Factor
HIF-1α: Hypoxia Inducible Factor-1
HK2: Hexokinase II
HO-1: Heme oxygenase-1
HREs: Hypoxia response elements
IFN-γ: Interferon-γ
IKK: Inhibitor of kappa B kinase
IL: Interleukin
JAK: Janus kinase
LPS: Lipopolysaccharide
MAPK: Mitogen-activated protein kinase
MCETs: Mast cell extracellular trap
MODS: Multiple organ dysfunction syndrome
mRNA: Messenger ribonucleic acid
mTOR: Mammalian target of rapamycin
NET: Neutrophil extracellular trap
NF-κB: Nuclear Factor-κB
NO: Nitric oxide
Nrf2: Nuclear factor erythroid 2-related factor 2
N-TAD: N-transactivation domain
ODDD: Oxygen-dependent degradation domain
PAS: Per-ARNT-Sim
PD-L1: Programmed death-ligand 1
PHDs: Prolyl hydroxylases
PI3K/Akt: Phosphatidylinositol 3-kinase/protein kinase B
PIP3: Inositol triphosphate
PKM2: Pyruvate kinase isozyme type M2
RORγt: Retinoid-related orphan nuclear receptor γt
ROS: Reactive oxygen species
SALI: Sepsis-associated acute lung injury
STAT3: Signal transducer and activator of transcription 3
TCR: T-cell receptor
TIIA: Tanshinone IIA
TLR: Toll-like receptor
TNF-α: Tumor necrosis factor-α
TR-AM: Tissue-resident alveolar macrophages
VEGF: Vascular endothelial growth factor
VHL: Von Hippel-Lindau
